# Monomethylhydrazine: MAK Value Documentation, addendum – Translation of the German version from 2021

**DOI:** 10.34865/mb6034e9_4ad

**Published:** 2024-12-23

**Authors:** Andrea Hartwig, MAK Commission

**Affiliations:** 1 Institute of Applied Biosciences. Department of Food Chemistry and Toxicology. Karlsruhe Institute of Technology (KIT) Adenauerring 20a, Building 50.41 76131 Karlsruhe Germany; 2 Permanent Senate Commission for the Investigation of Health Hazards of Chemical Compounds in the Work Area. Deutsche Forschungsgemeinschaft, Kennedyallee 40, 53175 Bonn, Germany. Further information: Permanent Senate Commission for the Investigation of Health Hazards of Chemical Compounds in the Work Area | DFG

**Keywords:** monomethylhydrazine, central nervous system, carcinogenicity, mutagenicity, toxicity, DNA methylation

## Abstract

The German Commission for the Investigation of Health Hazards of Chemical Compounds in the Work Area (MAK Commission) has re-evaluated the occupational exposure limit value (maximum concentration at the workplace, MAK value) of monomethylhydrazine [60-34-4] considering all toxicological end points. Relevant studies were identified from a literature search. The acute toxicity of monomethylhydrazine is caused by depletion of gamma-aminobutyric acid leading to the effects on the central nervous system. Chronic and subchronic exposure induces haemolytic effects and adverse effects on the liver. In carcinogenicity studies, inhaled monomethylhydrazine caused tumours of the lungs, liver, blood vessels and olfactory epithelium in female mice and tumours of the nose and adrenal glands in male hamsters. Orally applied, it induced tumours of the lungs and liver in mice and tumours of the colon and small intestine in hamsters. Monomethylhydrazine therefore remains classified in Carcinogen Category 2 and no MAK value can be derived. Monomethylhydrazine is genotoxic in vitro and in vivo and demonstrates a mutagenic potential in vitro. The substance forms methyl DNA adducts in vivo. On the basis of these effects and its structural similarity to 1,1-dimethylhydrazine and 1,2-dimethylhydrazine, which are genotoxic in germ cells, monomethylhydrazine has been classified in Germ Cell Mutagenicity Category 3 B. Monomethylhydrazine is irritating to the skin of dogs, guinea pigs and rabbits and to the eyes of rabbits. Although no valid studies considering dermal absorption are available, the “H” designation (for substances which can be taken up via the skin in toxicologically relevant amounts) has been retained because of the low dermal LD_50_ values in animals. Although no studies considering its sensitizing potential are available, the “Sh” designation (for substances which cause sensitization of the skin) has been retained because of its structural similarity to the known contact allergen hydrazine.

**Table:** 

**MAK value**	**–**
**Peak limitation**	**–**
	
**Absorption through the skin (1994)**	**H**
**Sensitization (1994)**	**Sh**
**Carcinogenicity (2020)**	**Category 2**
**Prenatal toxicity**	**–**
**Germ cell mutagenicity (2020)**	**Category 3 B**
	
**BAT value**	**–**
	
Synonyms	hydrazomethane
Chemical name (IUPAC)	methylhydrazine
CAS number	60-34-4
Structural formula	H_3_C–NH–NH_2_
Molecular formula	CH_6_N_2_
Molar mass	46.07 g/mol
Melting point	–52 °C (IFA 2019)
Boiling point at 1013 hPa	87.5 °C (US EPA 2010)
Density at 20 °C	0.88 g/cm^3^ (IFA 2019)
Vapour pressure at 25 °C	66.66 hPa (NCBI 2019)
log K_OW_	–1.05 (IFA 2019)
Solubility	miscible with water (NCBI 2019)
pKa value	7.87 at 30 °C (NCBI 2019)
**1 ml/m^3^ (ppm) ≙ 1.912 mg/m^3^**	**1 mg/m^3^ ≙ 0.523 ml/m^3^ (ppm)**
	
Hydrolytic stability	no data
Production	reaction of monochloramine and monoethylamine (Kennedy 2012)
Uses	solvent, intermediate and rocket fuel (ATSDR 1997)

Documentation for monomethylhydrazine was published in 1973 (Henschler 1973, available in German only). In this addendum, the substance and particularly its potential carcinogenic effects are re-evaluated on the basis of the more extensive data from studies that were carried out since 1972.

## 1 Toxic Effects and Mode of Action

Monomethylhydrazine causes irritation of the skin of dogs, rabbits and guinea pigs. In addition, it causes irritation of the eyes in rabbits.

After long-term inhalation exposure to monomethylhydrazine in a concentration of 2 ml/m^3^, lung adenomas, liver adenomas, liver carcinomas and haemangiomas developed in female mice; in addition, a low incidence of epithelial neoplasms of the olfactory and respiratory nasal mucosa were observed. Benign tumours in the nose and adrenal glands were found in male hamsters after exposure to a concentration of 2 ml/m^3^. In long-term studies with oral administration of 28.4 and 22 mg/kg body weight and day in the drinking water, the incidences of lung adenomas, bile duct angiomas and carcinomas and various liver tumours with shortened latency periods were increased in male and female mice, but not with statistical significance. In addition, histiocytomas and tumours in the caecum and colon developed in male and female hamsters after administration with the drinking water of 8.8 and 11.8 mg/kg body weight and day, respectively.

Haemolytic effects (in dogs, mice and monkeys), liver damage (in hamsters, mice and dogs) and lesions of the lymph nodes (in mice and dogs) and nasal mucosa (in hamsters and mice) were reported after chronic and subchronic exposure.

Valid studies of developmental toxicity are not available.

It seems likely that monomethylhydrazine has a skin sensitizing potential because hydrazine is a strong contact allergen and cross-reactions between hydrazine derivatives have been observed (Greim 1999). However, studies that specifically investigated monomethylhydrazine are not available.

Even though the numerous studies that are available are older and were not carried out according to current test guidelines, it is possible to draw the following conclusions with respect to genotoxicity: Monomethylhydrazine has mutagenic and clastogenic effects in bacteria and mammalian cells in vitro. Studies carried out in vivo yielded evidence of DNA-damaging effects including DNA adduct formation (covalent binding of methyl groups to DNA bases).

## 2 Mechanism of Action

### 2.1 Neurotoxicity

Neurological damage is one of the acute toxic effects that are caused by monomethylhydrazine. In animal studies, tremor, vomiting and convulsions were observed after administration of lethal and near lethal doses (NIOSH 1978; Sopher et al. 1968; Thomas and Young 2001). It was demonstrated that the substance initially acts on the amygdala of the brain and subsequently affects the limbic system (Shouse and Sterman 1979). The binding of monomethylhydrazine to carbonyl groups of vitamin B6 leads to the formation of hydrazones; as a result, pyridoxal phosphokinase may be inhibited causing a depletion of vitamin B6 and then of γ-aminobutyric acid (GABA) (Andersson et al. 1995; George et al. 1982). In mammals, GABA is a neurotransmitter of inhibitory synapses and decisive for the functioning of the nervous system. Therefore, binding to vitamin B6 derivatives contributes to the nerve-damaging effects of the substance and was likewise described as a toxic effect of 1,1-dimethylhydrazine (ATSDR 1997). Pyridoxine hydrochloride prevents the neurological symptoms caused by monomethylhydrazine (Andersson et al. 1995).

### 2.2 Genotoxicity and carcinogenicity

Genotoxic and carcinogenic effects may be caused by highly reactive intermediates that are formed during metabolism. In vivo studies yielded evidence that methyl groups bind to the DNA base guanine (N7 and O6) following exposure to monomethylhydrazine (Section 5.6). Methylation of guanine at the N7 and C8 positions was detected in vitro; the binding at N7 and at the C8 position indicates that the methyl diazonium ion and the methyl radical, respectively, are the alkylating intermediates (Augusto et al. 1990). Binding of the methyl group at the N7 and O6 positions of guanine may lead to DNA mismatches. There may be a close association between DNA methylation and the carcinogenic effects of the substance.

### 2.3 Effects on blood

The incubation of human blood samples with monomethylhydrazine led to the formation of methaemoglobin and Heinz bodies (agglutination of haemoglobin) (George et al. 1978). In other in vitro studies, red blood cells obtained from human blood were exposed to monomethylhydrazine in phosphate buffer in concentrations of 0.1 mM, 1 mM and 10 mM for periods of 2, 4 and 6 hours. After 2 hours, a methaemoglobin level of 22% was determined at the high concentration; however, it decreased over the course of incubation. Two hours after exposure, Heinz bodies had formed in nearly all cells; their numbers increased with time and concentration. At the high concentration, glutathione levels were markedly decreased 4 hours after exposure, but had returned to normal after 6 hours. The Heinz bodies decreased red cell deformability, caused cell deformation and destroyed erythrocytes (George 1975; Weinstein et al. 1975). In addition, red blood cells may be damaged by the oxidation of components of their cellular membrane, leading to an impairment of function (Schrier and Mohandas 1992).

### 2.4 Local irritation

Monomethylhydrazine causes severe irritation of the nose, skin and eyes (Kennedy 2012) because of its alkaline properties in aqueous solution (NIOSH 1978).

## 3 Toxicokinetics and Metabolism

### 3.1 Absorption, distribution, elimination

Data from human studies are not available.

Groups of 20 mice and 20 rats, 17 dogs and 16 monkeys were given single intraperitoneal injections of ^14^C-mono­methylhydrazine in doses of 22 mg/kg body weight (mice), 15 mg/kg body weight (rats) and 10 mg/kg body weight (dogs and monkeys). Radioactivity was determined in the tissues 2, 4, 8 and 24 hours after administration of the substance. Colorimetric tests were performed to determine the amount of starting substance present in the blood and urine samples. The highest concentrations in the tissues were found in mice and dogs after 4 hours and in monkeys after 2 hours. In rats, the concentrations in the tissues were not markedly increased at any time. The highest amounts of radioactivity were found in the liver, kidneys, bladder, pancreas and blood serum. In all species, the levels of radio­activity had decreased markedly after 24 hours. During the first 2 hours after substance administration, the amounts of radioactivity excreted with the urine by rats, mice and monkeys were twice as high as those excreted by dogs. After 24 hours, the levels of radioactivity excreted with the urine were 26% in dogs, 31% in monkeys, 40% in rats and 9% in mice. The amounts of radioactivity in the faeces or exhaled air were not determined. The starting substance made up about 50% of the excreted radioactivity at all sampling times (RAC 2014).

In another study, Sprague Dawley rats were given intraperitoneal injections of ^14^C-monomethylhydrazine in doses of 5.5, 11 or 22 mg/kg body weight. About 37%, 31% and 24% of the administered radioactivity was exhaled during the first 27 hours. The exhaled amount was found to consist of 20% to 25% ^14^CO_2_ and 75% to 80% ^14^CH_4_. In addition, in the dose groups that were given 5.5, 11 and 22 mg/kg body weight, about 41%, 39% and 22%, respectively, of the administered radioactivity had been excreted with the urine after 27 hours (Dost et al. 1966). According to the authors, the remaining amounts of administered radioactivity were still bound in the tissues and were determined after subtracting the amounts detected in the urine and exhaled air.

After 24 hours, female NMRI mice that were given subcutaneous injections of ^14^C-monomethylhydrazine in a dose of 15 mg/kg body weight had eliminated 36% of the injected radioactivity with the urine and 7% as ^14^CO_2_. After 3 hours, radioactivity was no longer detected in the blood of the animals. Male Wistar rats that were given subcutaneous injections of ^14^C-monomethylhydrazine in a dose of 5 mg/kg body weight had excreted 8% of the injected radioactivity with the bile after 24 hours (Hawks and Magee 1974).

Monomethylhydrazine was detected in the arterial blood only 30 seconds after non-occlusive application of the undiluted substance by means of a glass rod to the skin of the chest (300 cm^2^ shaved area of skin) of male anaesthetized dogs (16 crossbreeds) in doses of 0.32 to 5.75 mmol/kg body weight (14.7 to 265 mg/kg body weight). The animals were placed under a fume hood to avoid additional absorption of the substance by the lungs (Smith and Clark 1969). However, it remains unclear whether this actually prevented absorption by inhalation.

### 3.2 Metabolism

**Fig.1:**
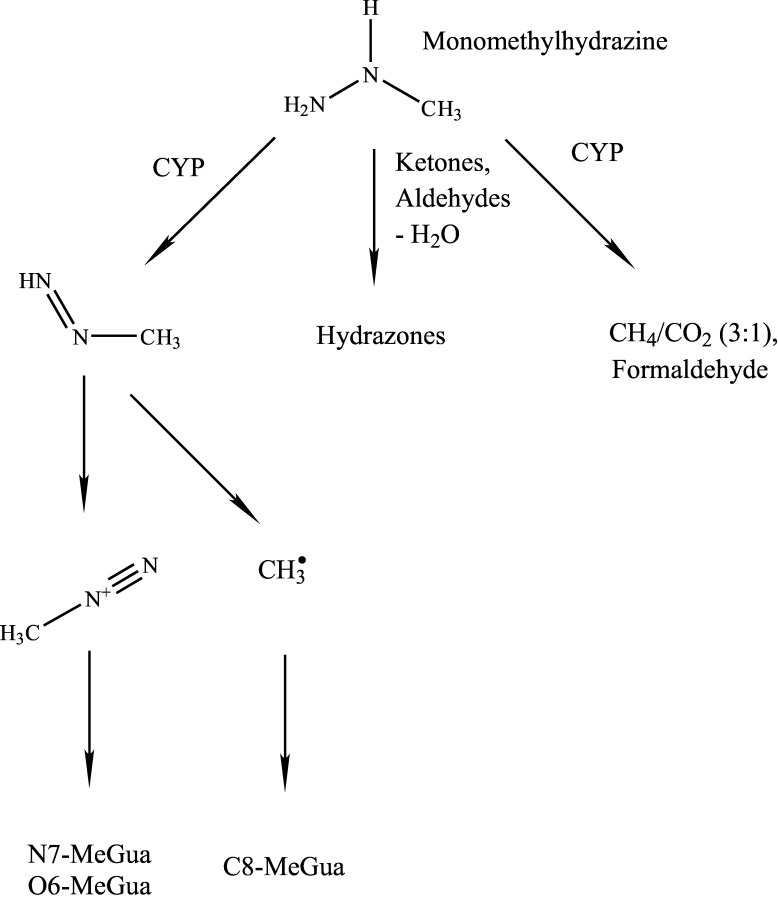
Transformation products of monomethylhydrazine; CYP: cytochrome P450

Monomethylhydrazine is degraded mainly in the liver via the oxidative pathway by means of monooxygenases to yield methyl radicals, methane, formaldehyde and carbon dioxide. Carbon dioxide and reactive metabolites formed in liver tissue sections of Sprague Dawley rats and formaldehyde formed with rat liver microsomes (Godoy et al. 1983; Wittkop et al. 1969) or S9 fractions (Godoy et al. 1983). Free methyl radicals were produced by cytochrome P450 in isolated hepatocytes and with liver microsomes from rats (Albano et al. 1993; Kalyanaraman and Sinhat 1985; Tomasi et al. 1987). In addition, the formation of methyl radicals from monomethylhydrazine was described in mouse fibroblasts (Avendaño and Menéndez 2008; Gamberini et al. 1998). Monomethylhydrazine reacts with aldehydes and ketones to form hydrazones (Andersson et al. 1995). Monomethylhydrazine is converted to methyldiazene via horseradish peroxidase; this leads to the formation of methyl radicals and methyldiazonium ions, which generate the DNA adducts C8-methylguanine and N7-methylguanine or O6-methylguanine (Augusto et al. 1990). Figure 1 shows the various metabolic pathways in the metabolism of monomethylhydrazine.

#### 3.2.1 Summary

After absorption, monomethylhydrazine is distributed in the organism and reaches mainly the liver, kidneys and bladder. The substance is transformed primarily by cytochrome P450 of the liver cells to yield methane, formaldehyde and carbon dioxide. During metabolism, methyl radicals and electrophilic intermediates such as the methyl diazonium ion may form as highly reactive intermediates.

## 4 Effects in Humans

### 4.1 Single exposures

The odour threshold for monomethylhydrazine is between 1.75 and 5.25 mg/m^3^. It has a fishy smell similar to that of ammonia (Kennedy 2012; Ruth 1986).

Five male test persons were exposed in a chamber to monomethylhydrazine in a concentration of 90 ml/m^3^ (170 mg/m^3^) for 10 minutes. The test persons inserted their heads into the exposure chamber through a membrane. The average age of the volunteers was 31 years and the group of test persons included active smokers, ex-smokers and non-smokers. Blood and urine samples were taken for analysis from all test persons before exposure and 1 day, 7 days and 14 days after exposure. Respiratory parameters were determined before and after exposure and 60 days after exposure. All test persons reported irritation of the nose and eyes and the perception of a pronounced smell. Increased moisture in the eyes without lacrimation and slight redness of the eyes were observed in most test persons. Each test person was then exposed to ammonia at a concentration of either 30 or 50 ml/m^3^; the aim was to compare the irritation and odour perceived. Exposure to monomethylhydrazine in a concentration of 90 ml/m^3^ (170 mg/m^3^) caused more severe irritation than exposure to ammonia in a concentration of 30 ml/m^3^, but the irritation was less severe than after exposure to an ammonia concentration of 50 ml/m^3^. The blood samples were analysed for electrolytes, the levels of calcium, phosphorus, cholesterol, bilirubin, protein, glucose, creatinine, chloride and blood urea nitrogen, the activities of lactate dehydrogenase and aspartate aminotransferase and the number of erythrocytes, leukocytes and Heinz bodies. Monomethylhydrazine had no effects on blood parameters. Seven days after exposure, Heinz bodies were observed in 3% to 5% of the red blood cells; however, their numbers decreased and they were no longer detected 60 days after exposure (MacEwen et al. 1970).

In an unpublished study, 42 NASA and Lockheed employees inhaled monomethylhydrazine in a single breath in a concentration of 0.38 mg/m^3^ (0.2 ml/m^3^). The substance was injected into a face mask in a volume of 30 cm^3^. Two thirds of the test persons perceived the odour of the substance and the substance caused irritation. Two hours after exposure, 75% reported itching in the nose. Vesiculation and cavities in the nasal mucosa were found in 12 of 42 test persons, and 4 of these 12 test persons additionally had slight bleeding of the nasal mucosa. There was no correlation between the occupational exposure of the test persons and either the occurrence of the signs and symptoms described or the ability to perceive the odour (Thomas and Young 2001). However, this study cannot be used for the evaluation due to the lack of a control group. The original study report from 1976 is not available in spite of efforts by the US EPA (2010) to obtain a copy. The reasons for the discrepancy between the results of this study and those of MacEwen et al. (1970), who reported findings of only slight irritation after exposure to far higher concentrations, are unclear.

### 4.2 Repeated exposure

The epidemiological data for the exposure of workers at rocket engine test facilities are shown in Section 4.7.

### 4.3 Local effects on skin and mucous membranes

One of the studies described in Section 4.1 reported findings of irritation of the eyes and nose after exposure to monomethylhydrazine in a concentration of 90 ml/m^3^.

### 4.4 Allergenic effects

It seems to be likely that monomethylhydrazine has a skin sensitizing potential because hydrazine is a strong contact allergen and cross-reactions between hydrazine derivatives are known (Greim 1999). However, specific studies for monomethylhydrazine are not available.

### 4.5 Reproductive and developmental toxicity

There are no data available.

### 4.6 Genotoxicity

Chromosomal changes were detected in the leukocytes of cancer patients who were treated with monomethylhydrazine as a cytostatic drug (Kimball 1977; Marinone and Venturelli 1970).

### 4.7 Carcinogenicity

Exposure to hydrazine increased the incidences of lung cancer and colorectal tumours in the workers of a rocket engine test facility in the United States. The workers (6044 in the cohort that was observed for mortality with 600 cases of cancer and 5049 in the cancer incidence cohort with 691 cases of cancer) were exposed to hydrazine and additionally to monomethylhydrazine and 1,1-dimethylhydrazine from 1950 to 1993 while handling rocket fuels. A job-exposure matrix was used to assess the exposure of the workers as low (control group, n = 3401 and 2800 workers in the mortality and cancer incidence cohorts, respectively), medium (n = 1593 and 1394 workers in the mortality and cancer incidence cohorts, respectively) and high (n = 1050 and 850 workers in the mortality and cancer incidence cohorts, respectively). If a lag phase of 20 years was taken into account, the relative risk for lung cancer, based on incidence, was 1.18 (95% confidence interval (CI): 0.62 to 2.24) and 2.49 (95% CI: 1.28 to 4.86) in the medium and high exposure groups, respectively, compared with the level of risk determined for the low exposure group. The dose–response trend was statistically significant. Smoking status was established only for some workers. According to the authors, smoking was not a confounder because they assumed that there were no differences in smoking status among the examined groups and the risks for other cancer sites associated with smoking were not increased. In both exposure groups, relative risks of 1.75 (95% CI: 0.93 to 3.30) and 2.16 (95% CI: 1.02 to 4.59) were obtained for tumours of the colon and rectum, respectively. The dose–response trend for these tumours was also statistically significant. Even without taking a lag phase into account, the relative risks for these sites were similar and statistically significant in the high exposure group (Ritz et al. 2006).

Another study of this cohort found no increase in mortality for lung cancer (0.89 (95% CI: 0.78 to 1.02)) or colorectal carcinomas (0.97 (95% CI: 0.75 to 1.22)) in the entire cohort. The relative risks for 315 test stand mechanics assessed as probably exposed to hydrazines were 1.67 (95% CI: 0.54 to 3.89) for colorectal carcinomas and 1.45 (95% CI: 0.81 to 2.39) for lung cancer (Boice et al. 2006).

The two studies used different methods to evaluate the data of the cohorts in order to estimate exposure levels. Whereas Ritz et al. (2006) assigned 2643 of 6044 persons to categories with medium or high exposure to hydrazine, Boice et al. (2006) assumed that based on 8372 persons who were examined with regard to mortality only 315 of a total of 1642 test stand mechanics had probable exposure to hydrazine. Boice et al. (2006) reported exposure mainly to monomethylhydrazine. However, in general it can be assumed, or at least not ruled out, that there was exposure to a mixture of hydrazine, 1,1-dimethylhydrazine and monomethylhydrazine. Therefore, the results support the assumption that the substance causes carcinogenic effects in humans; however, the data obtained are not sufficiently reliable to validate this assumption.

### 4.8 Other effects

Mushrooms belonging to the group of ascomycetes, such as the false morel, contain the toxin gyromitrin (*N*-methyl-­­*N*-formyl-*N*-acteyl hydrazone). After consumption by humans, gyromitrin is converted into *N*-methyl-*N*-formyl­hydrazine and acetaldehyde in the stomach. *N*-Methyl-*N*-formylhydrazine is hydrolytically decomposed in the ­stomach yielding monomethylhydrazine and formic acid. In humans, the symptoms of mushroom poisoning are headaches, vomiting, convulsions, damage to the liver and blood and respiratory failure; these are caused by the degradation products *N*-methyl-*N*-formylhydrazine and monomethylhydrazine (Andersson et al. 1995; Patočka et al. 2012). In vitro studies revealed severe changes in the cellular metabolism and the inhibition of cellular respiration after exposure of human HepaRG cells to monomethylhydrazine. These disturbances were attributed to the interaction of the ­intermediates that formed with the mitochondrial respiratory chain and to the inhibition of aminotransferase; these effects were described as being similar to those caused by hydrazine (Guyot et al. 2018).

## 5 Animal Experiments and in vitro Studies

### 5.1 Acute toxicity

#### 5.1.1 Inhalation

The LC_50_ values after inhalation exposure were 74 to 78 ml/m^3^ (4 hours) for rats, 56 to 65 ml/m^3^ (4 hours) for mice, 82 ml/m^3^ (1 hour) for monkeys, 143 ml/m^3^ (4 hours) for golden hamsters and 96 ml/m^3^ (1 hour) for dogs (Kennedy 2012).

#### 5.1.2 Oral administration

The LD_50_ values after oral administration were 32 mg/kg body weight for rats, 29 mg/kg body weight for mice (Kennedy 2012) and 22 mg/kg body weight for hamsters (Patočka et al. 2012).

#### 5.1.3 Dermal application

The LD_50_ values after dermal application were 93 mg/kg body weight for rabbits, 47 mg/kg body weight for guinea pigs, 239 mg/kg body weight for hamsters and 183 mg/kg body weight for rats (Kennedy 2012).

Undiluted monomethylhydrazine was applied non-occlusively by means of a glass rod to the skin of the chest (300 cm^2^ area of shaved skin) of anaesthetized dogs (16 crossbreeds) in doses of 14.7 to 265 mg/kg body weight, leading to erythema and oedema in addition to methaemoglobinaemia and convulsions at the high doses. The animals were placed under a fume hood to avoid additional absorption of the substance by the lungs (Smith and Clark 1969). However, it remains unclear whether this actually prevented absorption by inhalation.

#### 5.1.4 Intraperitoneal, subcutaneous and intravenous administration

The LD_50_ values for rats and mice were 21 and 15 mg/kg body weight, respectively, after intraperitoneal injection, 17 and 33 mg/kg body weight after intravenous injection and 35 and 25 mg/kg body weight after subcutaneous injection (Kennedy 2012). Dogs reacted with greater sensitivity than monkeys, rats and mice following a single intraperitoneal injection of a monomethylhydrazine dose of 10 mg/kg body weight. Kidney damage was observed (RAC 2014).

#### 5.1.5 Summary

The acute toxicity caused by monomethylhydrazine is characterized by convulsions, neurological effects, hypoglycaemia, vomiting, anaemia, increased bilirubin concentrations in the blood (liver damage), increased methaemoglobin levels and irritation of the nose and eyes (Kennedy 2012).

### 5.2 Subacute, subchronic and chronic toxicity

#### 5.2.1 Inhalation

A summary of the studies after repeated inhalation exposure is shown in Table 1. None of the reports specified the purity of the monomethylhydrazine used for exposure.

The studies used monkeys, dogs, rats, mice and hamsters as test animals. The target organs were mainly the blood, liver, kidneys, lymph nodes and, due to the local effects, the nasal epithelium. In mice, mortality was increased after exposure to monomethylhydrazine at concentrations of 2 ml/m^3^ and above. In rats, haemolysis was detected after exposure to monomethylhydrazine at the low concentration of 0.04 ml/m^3^ and above. The body weights of the rats were reduced even at the lowest concentration tested of 0.02 ml/m^3^. Therefore, a NOAEC (no observed adverse effect concentration) was not obtained.

**Tab.1:** Effects of monomethylhydrazine after repeated inhalation exposure

Species, strain, number per group	Exposure	Findings	References
dog, beagle, 4 ♂, 4 ♀	1 year, 0, 0.2, 2 ml/m^3^, 6 hours/day, 5 days/week, 7 years of observation	**0.2 ml/m^3^ and above**: Hct, Hb, RBC ↓; **2 ml/m^3^**: methaemoglobin level, BSP retention, ALT ↑; after 7 years of observation: no histopathological findings	MacEwen and Vernot 1978, 1985; US EPA 2010
hamster, Syrian golden, 200 ♂	1 year, 0, 0.2, 2, 5 ml/m^3^, 6 hours/day, 5 days/week	**0.2 ml/m^3^ and above**: body weights ↓; after observation for 52 weeks: **0.2 ml/m^3^ and above**: biliary cysts, submucosal cysts in the nasal epithelium and rhinitis; **2 ml/m^3^ and above**: nasal polyps, interstitial fibrosis of the kidneys, atelectasis; **5 ml/m^3^ and above**: inflammation of the liver	
rat, F344/N, 100 ♂, 100 ♀	1 year, 0, 0.02, 0.2, 2, 5 ml/m^3^, 6 hours/day, 5 days/week	**0.02 ml/m^3^ and above**: ♂: body weights ↓; **2 ml/m^3^ and above**: ♀: body weights ↓; after observation for 78 weeks: no histopathological findings	
mouse, C57BL/6J, 400 ♀	1 year, 0, 0.02, 0.2, 2 ml/m^3^, 6 hours/day, 5 days/week	after observation for 52 weeks: **0.02 ml/m^3^ and above**: inflammation, plasmocytosis and haemorrhages of the mandibular lymph nodes; **0.2 ml/m^3^ and above**: liver changes, cysts; **2 ml/m^3^ and above**: bile duct hyperplasia, hepatocellular pleomorphisms and gall stones; **5 ml/m^3^ and above**: angiectasis increased with statistical significance in the high concentration group	
monkey, no other details, 4 ♀	6 months, 0, 2, 5 ml/m^3^, 6 hours/day, 5 days/week	**2 ml/m^3^ and above**: no histopathological lesions in the heart, lungs, liver, spleen, kidneys	Kroe 1971
monkey, no other details, 4 ♂	a) 0.2 ml/m^3^, 6 months, 24 hours/day, 7 days/week b) 0.2 ml/m^3^, 145 days, 6 hours/day, 5 days/week c) 1 ml/m^3^, 144 days, 6 hours/day, 5 days/week d) control group	**in all exposed groups**: no histopathological lesions in the heart, lungs, liver, spleen, kidneys	
dog, beagle, 8 ♀	6 months, 0, 2, 5 ml/m^3^, 6 hours/day, 5 days/week	**2 ml/m^3^ and above**: cholestasis, haemosiderosis in the liver and kidneys	
dog, beagle, 8 ♂	a) 0.2 ml/m^3^, 6 months, 24 hours/day, 7 days/week b) 0.2 ml/m^3^, 145 days, 6 hours/day, 5 days/week c) 1 ml/m^3^, 144 days, 6 hours/day, 5 days/week d) control group	**in all exposed groups**: periportal, intracanalicular cholestasis, lymphoid hyperplasia in lymph nodes	
rat, Wistar, 10 ♂	6 months, 0, 2, 5 ml/m^3^, 6 hours/day, 5 days/week	**2 ml/m^3^ and above**: no histopathological lesions in the heart, lungs, liver, spleen, kidneys	
rat, Wistar, 10 ♂	a) 0.2 ml/m^3^, 6 months, 24 hours/day, 7 days/week b) 0.2 ml/m^3^, 145 days, 6 hours/day, 5 days/week c) 1 ml/m^3^, 144 days, 6 hours/day, 5 days/week d) control group	**in all exposed groups**: no histopathological lesions in the heart, lungs, liver, spleen, kidneys	
mouse, ICR, 10 ♂	6 months, 0, 2, 5 ml/m^3^, 6 hours/day, 5 days/week	**2 ml/m^3^:** **periportal cholestasis;** **2 ml/m^3^ and above**: bile duct proliferation, haemosiderosis in the liver, spleen and kidneys; **5 ml/m^3^**: centrilobular cholestasis, centrilobular haemosiderosis	
mouse, ICR, 10 ♂	a) 0.2 ml/m^3^, 6 months, 24 hours/day, 7 days/week b) 0.2 ml/m^3^, 145 days, 6 hours/day, 5 days/week c) 1 ml/m^3^, 144 days, 6 hours/day, 5 days/week d) control group	**in all exposed groups**: haemosiderosis in the liver, kidneys and spleen, more severe in groups a) and c)	
monkey, rhesus, 4 ♀	6 months, a) 0.2 ml/m^3^, 24 hours/day, 7 days/week b) 0.2, 1, 2, 5 ml/m^3^, 6 hours/day, 5 days/week c) control group	**in all exposed groups**: number of RBC ↓, formation of Heinz bodies	MacEwen and Haun 1971
dog, beagle, 8 ♀	6 months, a) 0.2 ml/m^3^, 24 hours/day, 7 days/week b) 0.2, 1, 2, 5 ml/m^3^, 6 hours/day, 5 days/week c) control group	**in all exposed groups**: RBC ↓, methaemoglobin level ↑, AP ↑, bilirubin in the serum ↑, formation of Heinz bodies	
rat, Wistar, 50 ♂	6 months, a) 0.2 ml/m^3^, 24 hours/day, 7 days/week b) 0.2, 1, 2, 5 ml/m^3^, 6 hours/day, 5 days/week c) control group	**2 ml/m^3^ and above**: body weight gains ↓	
mouse, ICR, 40 ♀	6 months, a) 0.2 ml/m^3^, 24 hours/day, 7 days/week b) 0.2, 1, 2, 5 ml/m^3^, 6 hours/day, 5 days/week c) control group	**2 ml/m^3^ and above**: mortality (15% and 27%) at 2 and 5 ml/m^3^	
monkey, rhesus, 4 ♀	90 days, 0, 0.04, 0.1 ml/m^3^, 24 hours/day, 7 days/week	no lesions	Darmer and MacEwen 1973
dog, beagle, 8 ♀	90 days, 0, 0.04, 0.1 ml/m^3^, 24 hours/day, 7 days/week	**0.1 ml/m^3^:** AP ↑, phosphorus in serum ↑, Hct, Hb, RBC ↓	
rat, Sprague Dawley, 80 ♂	90 days, 0, 0.04, 0.1 ml/m^3^, 24 hours/day, 7 days/week, examination after 45 and 90 days	**0.04 ml/m^3^ and above**: Hct, Hb, RBC ↓ (after 45 days), phosphorus ↑ (after 90 days); **0.1 ml/m^3^**: body weight gains ↓, RBC ↓ (after 90 days)	

ALT: alanine aminotransferase; AP: alkaline phosphatase; AST: aspartate aminotransferase; BSP: bromosulphthalein; Hb: haemoglobin; Hct: haematocrit; RBC: red blood cells

#### 5.2.2 Oral administration

See Section 5.7.2.

#### 5.2.3 Dermal application

There are no data available.

#### 5.2.4 Intraperitoneal injection

In a preliminary study, groups of 1 male and 1 female monkey (Macaca mulatta) were given daily single intraperitoneal injections of monomethylhydrazine in doses of 5 or 10 mg/kg body weight and day for 5 days. On day 2, vomiting was induced in the animals of both dose groups and continued to be observed in the animals given 10 mg/kg body weight after days 2 and 3. Convulsions began 3 to 5 days after the first dose was administered. The animals of this dose group died on day 5. The main study comprised two separate experiments. Experiment 1: Four male and 4 female monkeys were given daily intraperitoneal injections of monomethylhydrazine in a dose of 5 mg/kg body weight for 3 days, and 2 animals received saline injections throughout the experiment (control group). Subsequently, 2 of the males and 2 of the females were given 2.5 mg/kg body weight for 20 days, and the remaining 4 animals were given 2.5 mg/kg body weight for 8 days followed by 5 mg/kg body weight for 12 days. Vomiting and convulsions were transiently observed in both groups. Experiment 2: Three monkeys were given monomethylhydrazine injections in doses of 7 to 10 mg/kg body weight and day until they died. Two additional monkeys were used as a control group and received saline injections. Every other day, blood samples were taken and analysed. At the end of exposure, necropsies were performed on all animals. In this experiment, the animals died after 2, 3 or 4 days. Death was preceded by convulsions. The liver function was affected by exposure. A LOAEL (lowest observed adverse effect level) of 5 mg/kg body weight was established for monkeys on the basis of these test results (RAC 2014).

Another study investigated 10 female and 10 male monkeys (Macaca mulatta). The monkeys were divided into 5 groups. The control group was given saline injections for 14 days; the other groups were given either a single injection of monomethylhydrazine in a dose of 7.5 mg/kg body weight, daily injections of 2.5 mg/kg body weight for 14 days, injections of 5 mg/kg body weight every other day for 14 days or daily injections of 5 mg/kg body weight for 5 to 10 days. The renal function of each monkey was examined 24 hours after the injection of the final dose. All exposed animals showed signs of a loss of appetite and subsequently lost weight. In the group that received 5 mg/kg body weight every other day for 14 days, persistent vomiting was observed after the third injection and all became lethargic and weak. Convulsions were observed in 2 of the 4 animals. Changes in the morphology of the renal proximal and distal tubular cells were found in all exposed groups, but the renal function was not affected. A LOAEL of 2.5 mg/kg body weight was established for monkeys on the basis of these test results (RAC 2014).

### 5.3 Local effects on skin and mucous membranes

#### 5.3.1 Skin

In a study that investigated the acute toxicity in rabbits and guinea pigs, monomethylhydrazine was applied once occlusively to the shaved and bleached dorsal skin (Rothberg and Cope 1956). This led to slight oedema, which was reversible within 24 hours. In a study with dogs (16 crossbreeds), undiluted monomethylhydrazine was applied once to the skin of the chest (300 cm^2^ area of shaved skin) by means of a glass rod. The doses were 0.32 to 5.75 mmol/kg body weight (14.7 to 265 mg/kg body weight). This led to rapid discoloration of the skin, which persisted for 6 hours, and to oedema, which persisted up to 3 hours and was no longer detectable after 6 hours (Smith and Clark 1969).

REACH registrants classified monomethylhydrazine as corrosive to the skin (Skin Corr. 1B (H314)) (ECHA 2019) although these kinds of effects had not been documented by the available studies and the pKa value (of the conjugated acid) does not indicate high alkalinity.

#### 5.3.2 Eyes

In a study, 3 µl monomethylhydrazine was applied to the left eye of 10 rabbits. Conjunctivitis and erythema of the eyelids were observed 48 hours after application; the effects were reversible within 5 days (no other data; Rothberg and Cope 1956).

REACH registrants classified monomethylhydrazine in hazard class Eye Damage 1 (H318) based on the Skin Corr. 1B classification (ECHA 2019) although these kinds of effects had not been documented by the available studies and the pKa value (of the conjugated acid) does not indicate high alkalinity.

### 5.4 Allergenic effects

There are no data available.

### 5.5 Reproductive and developmental toxicity

#### 5.5.1 Fertility

Generation studies are not available.

Groups of 4 to 6 male BC3F1/CUM mice (11 to 18 weeks old) were given monomethylhydrazine in aqueous solution on 5 days by intraperitoneal injection at doses of 0, 7.5 or 12 mg/kg body weight and day. The doses corresponded to 25% and 40% of the LD_50_ of 30 mg/kg body weight, respectively. Sperm morphology and the number of sperm of the animals were analysed 3.5 weeks after exposure. A dose-dependent increase in the number of morphologically abnormal sperm was found compared with the number found in the control animals. Another 50 male BC3F1/CUM mice were given intraperitoneal injections of monomethylhydrazine in a dose of 3 mg/kg body weight and day for 5 days. Five mice were sacrificed and examined each week. An increased number of morphologically abnormal sperm (amorphous, subnormal, twin-tailed and folded) was observed; the values decreased and were similar to those in the control group after 6 weeks (Wyrobeck and London 1973).

#### 5.5.2 Developmental toxicity

The data from the reported studies are shown in Table 2.

The potential of monomethylhydrazine for developmental toxicity was investigated in F344 rats. In this study, 14 to 18 pregnant rats were given intraperitoneal injections of monomethylhydrazine in saline from days 6 to 15 of gestation. The doses were 0, 2.5, 5 and 10 mg/kg body weight and day. On day 20 of gestation, the test animals were sacrificed and the following parameters were determined: number and position of implantations, number of dead and live foetuses and number of resorptions. Other end points were foetal malformations (external, visceral and skeletal). The weight gains of the dams were reduced in relation to the dose compared with the weight gains in the vehicle control group. Likewise, convulsions were observed in 4 of 8 rats several times during the period of administration at 10 mg/kg body weight and day. At the low dose and above, there was a slight increase in the number of litters with more than 30% resorptions; however, this increase was not statistically significant. Visceral and skeletal variations including malformations were sporadically found in the foetuses. The authors reported that all effects were observed only at maternally toxic doses (Keller et al. 1984). Direct effects on the foetus induced by intraperitoneal injection of the substance cannot be ruled out. Therefore, the study has not been included in the evaluation.

Pregnant Sprague Dawley rats were used in another study; however, this study was not carried out according to currently valid guidelines. The animals were given daily intravenous injections of monomethylhydrazine in the following doses: saline (control group), 1.2 or 3.0 mg/kg body weight and day (low dose groups), 4.2 or 6.0 mg/kg body weight and day (medium dose groups) and 9.0 or 13.2 mg/kg body weight and day (high dose groups). The infusion rate was 10 µl/hour, and the animals were exposed from days 6 to 13 of gestation. The animals were sacrificed and examined on day 19 of gestation. Signs of maternal toxicity were observed at 9.0 mg/kg body weight and day and above. In the group exposed to 13.2 mg/kg body weight and day, 5 of 33 dams died before the end of the experiment, and convulsions were observed at 9 mg/kg body weight and above. The number of resorptions was increased in the medium and low dose groups; this increase was dose-dependent and statistically significant. In the groups treated with 3.0 and 4.2 mg/kg body weight and day, 70.6% and 100% of the dams, respectively, had at least one resorption. This was observed in 21.1% of the dams in the control group. The pregnancy rate was reduced with statistical significance at 6.0 mg/kg body weight and day and above. Only one dam was pregnant in the group that was given 6 mg/kg body weight and day, and 8 of 9 embryos were resorbed (89%) (Slanina et al. 1993). Teratogenicity was not examined.

In the second main experiment of the study, 16 pregnant Sprague Dawley rats were given single gavage doses of monomethylhydrazine on day 6 of gestation. The 24 animals of the control group were given saline and the exposure groups received monomethylhydrazine doses of 1 or 5 mg/kg body weight. On day 19 of gestation, the animals were sacrificed, the uterus was removed and the numbers of resorptions, live and dead foetuses, implantations and corpora lutea were determined. In the high dose group, the preimplantation loss per litter was increased with stat­istical significance based on the number of corpora lutea. It was 22.17% in the control group, 29.78% at 1 mg/kg body weight and 40.83% at 5 mg/kg body weight. The numbers of resorptions and dead foetuses were increased at 5 mg/kg body weight, but these increases were not statistically significant compared with the numbers in the control group (Slanina et al. 1993).

**Tab.2:** Studies of the reproductive toxicity of monomethylhydrazine

Sprague Dawley rats (14–18 rats per dose group and control group), intraperitoneal (Keller et al. 1984)												
Parameter	**Dose (mg/kg body weight and day)**											
	**0**		**2.5**				**5**				**10**	
weight gains of the dams^a)^												
days 6–10	8 ± 5		3 ± 3*				2 ± 4*				3 ± 3*	
days 11–16	21 ± 5		19 ± 10				15 ± 5				12 ± 8*	
days 17–20	30 ± 6		29 ± 10				25 ± 10				26 ± 12	
number of litters	13		15				15				16	
number of implantations/litter^a)^	7.8 ± 3.6		8.8 ± 3.4				7.3 ± 2.5				7.6 ± 3.4	
number of live foetuses/litter^a)^	6.8 ± 4.0		7.5 ± 3.4				6.2 ± 2.7				6.1 ± 3.9	
number of litters with more than 30% resorptions	0		2				3				3	
foetal weights (g)^a)^	3.1 ± 0.3		3.3 ± 0.3				3.1 ± 0.3				3.2 ± 0.3	
variations/malformations												
external^b)^	2 (2)		1 (1)				3 (4)				2 (2)	
visceral^b)^	1 (1)^c)^		2 (2)^d)^				6 (9)^d)^				3 (4)^e)^	
skeletal^b)^	0 (0)		1 (1)				1 (1)				2 (2)	

a) mean ± standard deviation

b) number of litters, number of animals in brackets

c) one foetus with anophthalmia and hydrocephalus

d) microphthalmia

e) hydronephrosis and dilated ureter in one foetus, hydrocephalus in 1 foetus and 2 foetuses with anophthalmia

*p < 0.05

**Table:** 

Sprague Dawley rats, intravenous (Slanina et al. 1993)												
Parameter	Dose (mg/kg body weight and day)											
	0	1.2		3.0		4.2		6.0		9.0		13.2
number of mated animals	22	21		22		28		15		6		33
number of dead dams	0	0		0		0		0		0		5
number of pregnant animals	19	16		17		16		1		0		0
number of animals with resorptions or no foetuses	3	5		5		12		14		6		28
Sprague Dawley rats, oral (Slanina et al. 1993)												
**Parameter**	**Dose (mg/kg body weight and day)**											
	**0**				**1**				**5**			
number of litters	24				16				16			
preimplantation loss/litter^a)^	22.17 ± 0.5				29.78 ± 0.4				40.83 ± 0.63*			
number of implantations^a)^	11.6 ± 3.8				10.9 ± 4.5				9.5 ± 4.1			
number of animals with resorptions	9 (37.5%)				3 (18.7%)				10 (62.5%)			
number of litters with malformations	2^b)^				1^c)^				0			
foetal weights (g)^a)^	2.39 ± 0.23				2.78 ± 0.41				2.61 ± 0.32			

a) mean ± standard deviation

b) 1 foetus with open eyes, 2 with skeletal malformations

c) 1 foetus with skeletal malformations

*p < 0.05

**Summary: **No teratogenic effects were observed in a developmental toxicity study with oral exposure of Sprague Dawley rats; however, this study was not carried out according to currently valid guidelines.

### 5.6 Genotoxicity

#### 5.6.1 In vitro

The data from in vitro studies that investigated genotoxicity are shown in detail in Table 3.

##### 5.6.1.1 Cell-free systems

Methylation of calf thymus DNA in the form of C8-methylguanine and N7-methylguanine was detected in the presence of monomethylhydrazine following enzymatic activation by horseradish peroxidase (Augusto et al. 1990).

##### 5.6.1.2 Bacteria and yeasts

Indicator tests with various Escherichia coli strains revealed possible DNA-damaging effects induced by monomethylhydrazine without metabolic activation (Poso et al. 1995; von Wright et al. 1977).

As pointed out in OECD Test Guideline 471 (OECD 2020), the Salmonella typhimurium strains TA1535, TA100, TA1537, TA97, TA1538 and TA98 do not provide reliable evidence of mutagenic effects that are caused by hydrazines. Therefore, the use of the Salmonella typhimurium strains TA102 and Escherichia coli WP2 is recommended for the detection of mutagenic effects induced by this group of substances. The mutagenicity tests available for monomethylhydrazine confirmed these findings. With a few exceptions, the studies with the Salmonella strains TA100, TA1537 and TA1538 yielded negative results. Two positive results and two negative results were obtained with the strain TA1535, and one negative result and one positive result were obtained with the strain TA98. In addition, no mutagenicity was detected using the strain TA97. Mutagenic effects were unequivocally induced by monomethylhydrazine in the strain TA102; all available studies yielded positive results. Mutagenicity was not found in a study in Escherichia coli WP2 (uvrA^–^) (Brusick and Matheson 1976), while tests with other WP2 mutants revealed mutagenic effects (von Wright et al. 1977; von Wright and Tikkanen 1980 a, b). Likewise, monomethylhydrazine yielded positive results in Escherichia coli ada mutants following activation by chemical oxidation (Sedgwick 1992).

The substance was not mutagenic in D4 yeast cells (Brusick and Matheson 1976).

##### 5.6.1.3 Mammalian cells

No DNA strand breaks were found in rat hepatocytes after alkaline elution (Sina et al. 1983). However, the validity of the study is questionable because the evaluation of cytotoxicity was not conclusive. Single strand breaks were induced by monomethylhydrazine in Ehrlich ascites tumour cells from ICR mice at concentrations of 0.4 mg/ml and above (Moroson and Furlan 1969). The UDS (DNA repair synthesis) test yielded evidence of DNA-damaging effects in rat and mouse hepatocytes. The tests were carried out with monomethylhydrazine sulfate (Mori et al. 1988). A UDS test in diploid human embryonic lung cells yielded negative results (Brusick and Matheson 1976).

Gene mutation tests in mammalian cells provided clear evidence of mutagenicity in the ouabain resistance test in hamster V79 lung fibroblasts (data from an abstract; Kuszynski et al. 1981); however, no mutagenic effects were detected in TK^+/–^ tests with mouse lymphoma cells (Brusick and Matheson 1976; Rogers and Back 1981)*.* Tests for mutations inducing resistance in systems with ouabain, thioguanine or cytosine arabinoside in mouse lymphoma cells yielded negative results (Rogers and Back 1981).

**Tab.3:** Studies of the genotoxicity of monomethylhydrazine in vitro

End point	Test system	Concentration [µg/plate]^a)^	Effective concentration [µg/plate]^a)^	Cytotoxicity [µg/plate]^a)^	Result		References
					–m. a.	+m. a.	
indicator tests differential killing bacteria	Escherichia coli WP2 trpE56, Escherichia coli CM871 trpE65 uvrA155 recA56 lexA	92–920	no data		+	n. t.	Poso et al. 1995
	Escherichia coli W 3110 try her^+^, Escherichia coli B/r WP2 try	500, 1000	1000		+	n. t.	von Wright et al. 1977
	Escherichia coli W 3110 thy polA_1_^+^, Escherichia coli W 3110 thy polA_1_	500, 1000	500		+	n. t.	von Wright et al. 1977
gene mutation Escherichia coli	Escherichia coli WP2 try hcr	0, 5, 10, 20 µg/ml	5 µg/ml	≥ 5 µg/ml	+	n. t.	von Wright et al. 1977
	Escherichia coli ada mutants, after chemical oxidation as the mechanism of activation	0, 0.5, 1.0, 1.5, 2.0 mM	0.5 mM	+	–	+	Sedgwick 1992
	Escherichia coli WP2 uvrA^– ^(spot test)	0, 1.0 µl/plate (–m. a.) 0, 5.0 µl/plate (+m. a.)	–	10 µl/plate	–	–	Brusick and Matheson 1976
	Escherichia coli WP2B/r trp, Escherichia coli WP2 uvrA trp, Escherichia coli CM871 uvrA rec A lexA trp (spot test)	0, 23, 46, 92	23 and above	no data	+	n. t.	von Wright and Tikkanen 1980 a
	Escherichia coli WP2B/r trp, Escherichia coli WP2 uvrA trp, Escherichia coli CM871 uvrA rec A lexA trp (modified preincubation test)	0, 23, 46	23 and above	no data	+	n. t.	
	Escherichia coli WP2 uvrA trp (spot test and modified preincubation test)	0, 23, 46, 92	23 and above	≥ 23	+	n. t.	von Wright and Tikkanen 1980 b
gene mutation Salmonella typhimurium	Salmonella typhimurium TA102 (preincubation test)	0, 23, 46, 92	23	no data	+	n. t.	Poso et al. 1995
	Salmonella typhimurium TA100 (preincubation test)	0–92 growth period before treatment: 11 hours	no data	no data	+	–	Matsushita et al. 1993
	Salmonella typhimurium TA102 (with modified preincubation method)	0–460 growth period before treatment: 5, 7 or 11 hours	no data	no data	+	–	
	Salmonella typhimurium TA1535 (preincubation test)	0, 100, 200, 500, 1000	500	≥ 500 < 50% surviving cells	+	+	Rogan et al. 1982
	Salmonella typhimurium TA1537 (preincubation test)	0, 100, 200, 500, 1000	1000	≥ 500 (–m. a.) 200 (+m. a.) < 50% surviving cells	–	+	
	Salmonella typhimurium TA100 (modified preincubation test)	0, 46, 92, 138	–	> 138	–	–	von Wright and Tikkanen 1980 b
	Salmonella typhimurium TA98 (spot test)	0, 92, 230, 460	460	–	+	n. t.	von Wright et al. 1978
	Salmonella typhimurium TA100 (spot test)		–	–	–	–	
	Salmonella typhimurium TA1950 (spot test)		–	–	–	–	
	Salmonella typhimurium TA100 (plate incorporation test)	0, 10, 50, 100, 200	–	no data	–	–	
	Salmonella typhimurium TA98 (spot test and plate incorporation test)	0, 0.0001, 0.001, 0.01, 0.1, 1.0 µl/plate (–m. a.) 0, 0.01, 0.1, 1.0, 5.0 µl/plate (+m. a.)	–	10 µl/plate	–	–	Brusick and Matheson 1976
	Salmonella typhimurium TA100 (spot test and plate incorporation test)		–	10 µl/plate	–	–	
	Salmonella typhimurium TA1535 (suspension test)		–	10 µl/plate	–	–	
	Salmonella typhimurium TA1537 (spot test and plate incorporation test)		–	10 µl/plate	–	–	
	Salmonella typhimurium TA1538 (spot test and plate incorporation test)		–	10 µl/plate	–	–	
	TA1535 (suspension test)	0, 1, 5 µl/ml	1 µl/ml		n. t.	+	
	Salmonella typhimurium TA98 (plate incorporation test)	0, 1, 5.5, 10, 33, 100	–	> 100	–	–	Mortelmans et al. 1986
	Salmonella typhimurium TA100 (plate incorporation test)		–	> 100	–	–	
	Salmonella typhimurium TA1535 (plate incorporation test)		–	> 100	–	–	
	Salmonella typhimurium TA1537 (plate incorporation test)		–	> 100	–	–	
gene mutation yeast	Saccharomyces cerevisiae D4 (spot test and plate incorporation test)	0, 0.0001, 0.001, 0.01, 0.1, 1.0, 5.0 µl/plate (–m. a.) 0, 0.01, 0.1, 1.0, 5.0 µl/plate (+m. a.)	–	10 µl/plate	–	–	Brusick and Matheson 1976
indicator tests mammalian cells	DNA single strand breaks (alkaline density gradient centrifugation), Ehrlich ascites tumour cells from mice ♀ (Ha/ICR)	0, 40, 120 µg/ml	40 µg/ml	–	+	–	Moroson and Furlan 1969
	DNA strand breaks (alkaline elution), rat hepatocytes	0, 0.03, 0.3, 3 mM	–	cytotoxicity not valid	–	–	Sina et al. 1983
	UDS, human embryonic lung cells (WI-38)	0, 0.1, 0.5, 1.0 µl/ml	–	–	–	–	Brusick and Matheson 1976
	UDS, rat hepatocytes (ACI)	0, 0.01–1 mM as methylhydrazine sulfate	0.1 mM	1 mM	+	n. t.	Mori et al. 1988
	UDS, mouse hepatocytes (C3H/HeN)	0, 0.1–1 mM as methylhydrazine sulfate	1 mM	not cytotoxic in the tested range	+	n. t.	
gene mutation mammalian cells	hprt test, hamster lung fibroblasts (V79)	no data	no data	no data	+	+	Kuszynski et al. 1981
	TK^+/–^ test, mouse lymphoma cells (L5178Y), selective system: thymidine excess	0, 0.1, 1, 2.5, 5 mM	–	≥ 1 mM	–	n. t.	Rogers and Back 1981
	mutation to ouabain, thioguanine or cytosine arabinoside resistance, mouse lymphoma cells (L5178Y)	0, 0.1, 1, 2.5, 5 mM	–	≥ 1 mM	–	n. t.	
	TK^+/–^ test, mouse lymphoma cells (L5178Y), selective system: bromodeoxyuridine	0, 0.0005, 0.001, 0.05, 0.1 µl/ml (–m. a.) 0, 0.001, 0.005, 0.01, 0.05 µl/ml (+m. a.)	–	no data	–	–	Brusick and Matheson 1976

–: negative result; +: positive result; m. a.: metabolic activation; n. t.: not tested; UDS: DNA repair synthesis test

a) unless otherwise specified

#### 5.6.2 In vivo

The data from in vivo studies that investigated genotoxicity are shown in detail in Table 4.

##### 5.6.2.1 Bacteria in vivo/ex vivo

Monomethylhydrazine did not induce mutagenic effects in a host-mediated assay (in vivo/ex vivo) with NMRI mice and in the Salmonella typhimurium strain TA1950. However, the authors attributed the negative results to the use of a strain lacking sensitivity and the presence of an insufficient number of bacteria in the peritoneal fluid as a result of bacteriotoxicity induced by monomethylhydrazine (von Wright et al. 1978). A follow-up study yielded weakly positive results. The authors attributed the marginal mutagenic activity to the large amounts of intact monomethylhydrazine in the peritoneal fluid and the bacteriotoxic effects (von Wright and Tikkanen 1980 b).

##### 5.6.2.2 Mammals

###### Indicator tests

Various indicator tests with mammals yielded positive results for monomethylhydrazine: N7-methylguanine DNA adducts were detected in the liver and colon of female NMRI mice that were given subcutaneous injections of ^14^C-monomethylhydrazine in a dose of 15 mg (Hawks and Magee 1974). In another study, Swiss mice were given a gavage dose of monomethylhydrazine of 15 mg/kg body weight and golden hamsters were given gavage doses of 0, 11.3, 14.1, 17.6, 22 or 27.5 mg/kg body weight. Here, only low-level binding of methyl groups to the bases of the DNA (N7-methylguanine and O6-methylguanine) was observed in the liver of mice. The tissues of mice were examined 1 hour after administration of the substance, and the liver tissues of hamsters were analysed after 24 hours (Bosan and Shank 1983). Administration by intraperitoneal injection for 5 days or with the drinking water for 14 days induced DNA methylation in the liver cells of Sprague Dawley rats (N7-methylguanine) or in the liver and kidney cells (O6-methylguanine in the liver and kidneys and N7-methylguanine in the liver) of BALB/c mice (Bergman and Hellenäs 1992). In addition, DNA single strand breaks were observed in Ehrlich ascites tumour cells that were cultured in ICR mice. The animals were given single intraperitoneal injections of monomethylhydrazine in doses of 10 or 20 mg/kg body weight (Moroson and Furlan 1969). The UDS test yielded negative results in hepatocytes from rats that were exposed once to a gavage dose of monomethylhydrazine (30 mg/kg body weight) (Beije and Olsson 1990).

###### Germ cells

A study of Wyrobeck and London (1973) that investigated sperm anomalies was described in detail in Section 5.5.1. In general, these studies require critical analysis because changes in sperm morphology are not reliable indicators of actual mutations and the relevance of these effects for germ cell mutagenicity is questionable (ICPEMC 1983; Salamone 1988; Wild 1984). Therefore, the results of the two studies described above can be interpreted only as cytotoxic effects and cannot be used for the evaluation of possible germ cell mutagenicity. Dominant lethal tests were carried out with 7 to 8-week-old ICR mice and 10 to 12-week-old Sprague Dawley rats. The mice were given monomethylhydrazine doses of 0, 0.26, 0.86, 2.6 or 26 mg/kg body weight and day. The rats were treated with 0, 0.215, 0.72 and 2.15 mg/kg body weight and day. Each group consisted of 10 male animals. Both species were given daily intraperitoneal injections for 5 days. Monomethylhydrazine did not induce clastogenic or aneugenic effects (Brusick and Matheson 1976). The study deviated from OECD Test Guideline 478 (OECD 2016) in so far as the number of treated animals was too small and the mating period of the animals was too short (7 weeks instead of 10 weeks). In addition, the highest dose was equivalent to 1/10 of the LD_50_ because of the toxicity of monomethylhydrazine. This dose may have been too low to detect clastogenic or aneugenic effects. For these reasons, the dominant lethal test has not been included in the evaluation of germ cell mutagenicity.

**Tab.4:** Studies of the genotoxicity of monomethylhydrazine in vivo

Test system	Species, organ/tissue	Dose	Results	Comments	References
**in vivo/ex vivo**					
host-mediated assay	mouse (NMRI) 3 ♂ per group, (Salmonella typhimurium TA1950)	0, 30 mg/kg body weight, gavage, single	–	bacteriotoxic	von Wright et al. 1978
	mouse (NMRI) 4 ♂ per group, (Salmonella typhimurium TA1950)	0, 33 mg/kg body weight, gavage, single	+		von Wright and Tikkanen 1980 b
**mammals**					
indicator tests	covalent DNA binding mouse (NMRI), 12 ♀, liver, colon, kidneys, isolation of the tissues 6 hours after treatment	15 mg/kg body weight, subcutaneous, single	+	N7-methylguanine: liver, colon	Hawks and Magee 1974
	covalent DNA binding mouse (Swiss Webster) 10 ♂, liver, isolation of the tissues 1 hour after treatment	15 mg/kg body weight, gavage, single	+	N7-methylguanine, O6-methylguanine: liver	Bosan and Shank 1983
	covalent DNA binding hamster (Syrian golden), 6 ♂ per group, liver, isolation of the tissues 24 hours after treatment	0, 11.3, 14.1, 17.6, 22, 27.5 mg/kg body weight, gavage, single	–	–	
	covalent DNA binding mouse (BALB/c), 10 ♂, liver, kidneys, lungs	7.5 mg/kg body weight, intraperitoneal, single	–	–	Bergman and Hellenäs 1992
	covalent DNA binding rat (SD), 1 ♂, liver, kidneys, lungs	6.4 mg/kg body weight, intraperitoneal, single	–	–	
	covalent DNA binding mouse (BALB/c), 10 ♂, liver, kidneys, lungs	13 mg/kg body weight and day, drinking water, 14 days	+	O6-methylguanine: liver and kidneys; N7-methylguanine: liver	
	covalent DNA binding rat (SD), 1 ♂, liver, kidneys, lungs	7.3 mg/kg body weight and day, intraperitoneal on 5 days	+	N7-methylguanine: liver	
	single strand breaks (alkaline density gradient centrifugation), Ehrlich ascites tumour cells in mice (ICR) ♀	0, 10, 20 mg/kg body weight, intraperitoneal, single	+	–	Moroson and Furlan 1969
	UDS test, rat, no other details, (study is available only as an abstract)	0, 30 mg/kg body weight, gavage, single	–	–	Beije and Olsson 1990
**germ cells**					
dominant lethal test	mouse (NMRI), 10 ♂ per group, each male given 2 females for a mating period of 5 days for 8 consecutive weeks; different females were used each week	0, 0.26, 0.86, 2.6 mg/kg body weight and day, intraperitoneal, 5 days	–	LD_50_: 26 mg/kg body weight, low fertility, questionable validity (see text)	Brusick and Matheson 1976
	rat (SD), 10 ♂ per group, each male given 2 females for a mating period of 5 days for 7 consecutive weeks; different females were used each week	0, 0.215, 0.72, 2.15 mg/kg body weight and day, intraperitoneal, 5 days	–	LD_50_: 21.5 mg/kg body weight, high mortality, questionable validity (see text)	

–: negative result; +: positive result

##### 5.6.2.3 Accessibility of the germ cells

Sperm anomalies were observed after intraperitoneal injection (Wyrobeck and London 1973). However, this route of exposure does not allow conclusions to be drawn about the accessibility of the germ cells via systemic distribution.

#### 5.6.3 Summary

There are a large number of earlier studies available. Although these do not comply with current test guidelines, the following conclusions may be drawn with respect to genotoxicity.

Monomethylhydrazine induced gene mutations in Escherichia coli strains. In the Salmonella mutagenicity test, the substance unequivocally caused mutagenicity mainly in the strain TA102. The negative results obtained in bacter­ial tests in vitro were probably due to the bacteriotoxic effects of the substance. In the studies described, the high concentrations were found to be too toxic for bacteria; analysis of the mutagenic effects was therefore not possible (von Wright and Tikkanen 1980 a). In addition, bacterial cells were more sensitive to monomethylhydrazine in preincubation tests than in plate incorporation tests (Rogan et al. 1982). In vivo studies yielded evidence of the covalent binding of methyl groups to DNA bases following exposure to monomethylhydrazine. Methylation of the N7 and O6 positions of guanine was detected and C8-methylguanine additionally formed in a cell-free system. The first two forms of methylation are probably due to the formation of methyldiazonium ions (Augusto et al. 1990; Sedgwick 1992), whereas C8-methylguanine formed from methyl radicals (Augusto et al. 1990). The substance was found to be clastogenic in vitro. Furthermore, in an hprt test in vitro, monomethylhydrazine induced mutagenic effects in somatic cells.

### 5.7 Carcinogenicity

#### 5.7.1 Short-term studies

CDF-1 mice (aged 7 to 8 weeks) were given monomethylhydrazine in aqueous solution once a week for 8 weeks either by gavage (females: 0.46 mg in 0.2 ml) or by intraperitoneal injection (males: 0.23 mg in 0.1 ml). The total dose was 3.7 mg/mouse (females) and 1.8 mg/mouse (males). On the basis of an average body weight in week 12 of 0.029 kg, this dose corresponded to weekly doses of 16 mg/kg body weight for female mice and 8 mg/kg body weight for male mice (2.2 and 1.1 mg/kg body weight and day, respectively). The control groups were given saline. The control and dose groups each consisted of 27 female and 31 male mice. By the end of the study, 70% of the exposed females and none of the exposed males had died. In the control groups, mortality after oral and intraperitoneal administration of saline was 0% and 10%, respectively. Up to week 32 after exposure, the tumour incidence in the exposed mice was not increased with statistical significance compared with that in the relevant control group (Kelly et al. 1969). This study cannot be included in the evaluation of the carcinogenic potential of monomethylhydrazine because the exposure and observation periods were too short.

#### 5.7.2 Long-term studies

##### 5.7.2.1 Inhalation

The data from studies that investigated the carcinogenic effects of monomethylhydrazine after inhalation are shown in Table 5.

An inhalation study with 1-year exposure to monomethylhydrazine was carried out in 10-week-old male and female F344/N rats, 10-week-old female C57BL/6J mice, 12-week-old male Syrian golden hamsters (LAK:LVG[SYR]) and 11 to 20-month-old female and male beagle dogs. Each concentration was tested in 100 male and 100 female rats and 150 control animals per sex. Per concentration, 400 female mice, 200 male hamsters and 4 dogs were used. The monomethylhydrazine concentrations were 0, 0.02, 0.2, 2 and 5 ml/m^3^ for rats, 0, 0.02, 0.2 and 2 ml/m^3^ for mice, 0, 0.2, 2 and 5 ml/m^3^ for golden hamsters and 0, 0.2 and 2 ml/m^3^ for dogs. Inhalation was carried out for 6 hours a day, on 5 days a week, for 52 weeks and was followed by an observation period lasting from 52 weeks to 7 years. The non-neoplastic findings are described in Section 5.2.1. The number of pituitary gland adenomas was increased with statistical significance in female rats, but the increase was not dependent on the concentration. Therefore, it is questionable whether these tumours were induced by monomethylhydrazine. In golden hamsters, the incidences of nasal polyps were increased at monomethylhydrazine concentrations of 2 ml/m^3^ and above and the incidences of nasal adenomas and cortical adenomas of the adrenal glands were increased at 5 ml/m^3^. After 43 weeks, the number of mice that survived in the control group and in the groups exposed to of 0.02, 0.2 and 2 ml/m^3^ was 356, 337, 349 and 361, respectively. In mice, the incidences of lung adenomas, liver adenomas and liver carcinomas were increased with statistical significance at a concentration of 2 ml/m^3^. In the high concentration group, osteomas, adenomatous polyps and epithelial neoplasms of the olfactory and respiratory nasal mucosa were found in some mice; these effects were not observed in any animal of the control group. Furthermore, the number of haemangiomas was increased with statistical significance in the high concentration group. In dogs, no tumours were induced by monomethylhydrazine (MacEwen and Vernot 1985).

Although the exposure period of the study of 1 year was only half as long as required by the current OECD Test Guideline 451 (OECD 2018) and fewer examinations were carried out, overall, the study provides evidence of carcinogenic effects that were induced by the substance in mice and hamsters following inhalation exposure.

**Tab.5:** Inhalation studies of the carcinogenicity of monomethylhydrazine

Author:	MacEwen and Vernot 1985								
Substance:	monomethylhydrazine								
Species:	**rat**, F344/N, 100 ♂, 100 ♀ per concentration, 150 ♂, 150 ♀ as control animals **mouse**, C57BL/6J, 400 ♀ per concentration and control group **Syrian golden hamster**, LAK: LVG[SYR], 200 ♂ per concentration and control group **dog**, beagle, 4 ♂, 4 ♀ per concentration and control group								
Administration route:	inhalation								
Concentration:	0, 0.02 (only rat and mouse), 0.2, 2, 5 (only rat and hamster) ml monomethylhydrazine/m^3^								
Duration:	1 year, 5 days/week, 6 hours/day, observation period: 52 weeks in mice and hamsters, 78 weeks in rats, 7 years in dogs								
Toxicity:	occasional non-neoplastic effects in all treated groups of animals; see Section 5.2.1								
**rats**			**Exposure concentration (ml/m^3^)**							
		**0**	**0.02**		**0.2**		**2**		**5**
surviving animals^a)^	♂	144	99		97		90		90
	♀	146	98		96		93		80
**tumours**						
pituitary adenomas	♂	44/150 (29%)	34/100 (34%)		32/100 (32%)		23/99 (23%)		18/99 (18%)
	♀	43/149 (29%)	45/99 (45%)**		43/100 (43%)**		48/99 (48%)**		26/99 (26%)
**mice**			**Exposure concentration (ml/m^3^)**							
		**0**		**0.02**		**0.2**		**2**	
surviving animals^a)^	♀	356		337		349		361	
**tumours**						
**nasal mucosa:**						
adenomas	♀	0/367 (0%)		1/354 (0.3%)		0/349 (0%)		1/355 (0.3%)	
adenomatous polyps	♀	0/367 (0%)		0/354 (0%)		0/349 (0%)		4/355 (1.1%)	
osteomas	♀	0/367 (0%)		0/354 (0%)		0/349 (0%)		3/355 (0.8%)	
epithelial neoplasms (nasal and respiratory mucosa)	♀	0/367 (0%)		2/354 (0.6%)		1/349 (0.3%)		4/355 (1.1%)	
**lungs:**						
adenomas	♀	13/364 (4%)		16/354 (5%)		23/347 (7%)		56/360 (16%)**	
carcinomas	♀	0/364 (0%)		1/354 (0.3%)		2/347 (0.6%)		3/360 (0.8%)	
**liver:**									
adenomas	♀	6/373 (2%)		2/357 (0.6%)		5/357 (1%)		20/363 (5.5%)**	
carcinomas	♀	2/373 (0.5%)		4/357 (1%)		4/357 (1%)		14/363 (4%)**	

haemangiomas	♀	5/387 (1%)		9/371 (2%)		5/368 (1%)		22/371 (6%)**	
haemangiosarcomas	♀	1/387 (0.3%)		4/371 (1%)		4/368 (1%)		5/371 (1%)	
**hamsters**			**Exposure concentration (ml/m^3^)**							
		**0**		**0.2**		**2**		**5**	
surviving animals^b)^	♂	187		184		166		151	
**tumours**									
**nose:**									
adenomas	♂	1/190 (0.5%)		0/177 (0%)		0/180 (0%)		7/177 (4%)*	
polyps	♂	0/190 (0%)		0/177 (0%)		9/180 (5%)**		11/177 (6%)*	
**adrenal glands:**	♂								
cortical adenomas (benign)	♂	16/191 (8%)		16/173 (9%)		10/172 (6%)		23/176 (13%)**	

* p ≤ 0.05

** p ≤ 0.01

a) mortality after 52 weeks

b) mortality after 43 weeks (MacEwen and Vernot 1978)

##### 5.7.2.2 Oral administration

The data from studies that investigated the carcinogenic effects of monomethylhydrazine after oral administration are shown in Table 6.

Monomethylhydrazine was administered to groups of 50 female and 50 male 6-week-old Swiss mice in the drinking water (0.01%) for their entire lifespan. The average daily consumption was 0.71 mg monomethylhydrazine per female mouse and 0.66 mg monomethylhydrazine per male mouse. Doses of 20.3 and 17.8 mg/kg and day were estimated on the basis of the assumed body weights of 0.035 kg for female mice and 0.037 kg for male mice, respectively (US EPA 2010). Control data from 110 control animals per sex that were obtained from an earlier study that investigated methylhydrazine sulfate were used for comparison (Toth 1969). Monomethylhydrazine reduced the survival of the mice. Half of the exposed female and male mice died after 45 and 30 weeks, respectively, whereas half of the female and male control animals died only after 100 and 80 weeks, respectively. Lung adenomas were detected in 24% of the exposed female mice and 22% of the exposed male mice. In both groups, the average latency period was 51 weeks. A number of benign and malignant liver cell tumours and bile duct angiomas and carcinomas were observed in both sexes; these did not develop in the control groups. Few other neoplasms were found; however, the number of these tumours was increased with statistical significance compared with the numbers detected in the control group. The latency periods for the tumours that occurred were shorter compared with those in the control group (Toth 1972).

In another study, monomethylhydrazine was administered to groups of 50 female and 50 male 6-week-old golden hamsters in the drinking water (0.01%) for their entire lifespan. The average daily consumption of monomethylhydrazine was 1.3 mg in female golden hamsters and 1.1 mg in male golden hamsters. Doses of 9.0 and 8.2 mg/kg body weight and day were estimated on the basis of the assumed body weights of 0.145 kg for female hamsters and 0.134 kg for male hamsters, respectively (US EPA 2010). The control group consisted of 100 female and 100 male golden hamsters. Ten weeks after the beginning of the study, the survival of the animals was reduced by the administered dose with statistical significance compared with that of the control group. Malignant histiocytomas of the liver (Kupffer cell sarcomas) were observed in 32% of the exposed female golden hamsters and 54% of the exposed male golden hamsters. The average latency periods were 70 and 78 weeks, respectively. These tumours were not found in any control animal. Furthermore, 9 exposed female hamsters (18%) developed various tumours in the caecum (adenomas and adenocarcinomas) with a latency period of 64 weeks. These tumours of the caecum were likewise observed in 7 exposed male hamsters with a latency period of 77 weeks. In addition, other tumours were observed. However, their incidences were lower and not increased with statistical significance compared with those in the control groups (Toth and Shimizu 1973).

The described study was repeated to take possible hydrolytic degradation of monomethylhydrazine in the drinking water into account. Monomethylhydrazine was administered to 5-month-old male golden hamsters in the drinking water (0.01%) for their entire lifespan. Two groups of 30 animals were given monomethylhydrazine in untreated drinking water (7.3 mg/kg body weight and day, pH not reported) or in drinking water adjusted with HCl to a pH of 3.5 (7.5 mg/kg body weight and day). A control group of 17 animals was given drinking water adjusted with HCl to a pH of 3.5. After 90 weeks, survival was 12% in the animals of the control group and 3% in the group that was given methylhydrazine in the drinking water with a pH that had not been adjusted; no animals survived in the group that received methylhydrazine in drinking water adjusted to pH 3.5. After 3 months, the body weights of hamsters that were given drinking water buffered with monomethylhydrazine were decreased. After 11 months, all groups had reduced body weights. Blood samples were taken from 5 animals of each group after 7, 11 and 15 months, and all animals were evaluated histopathologically at the end of the study. The haematocrit and the number of red blood cells were reduced in both dose groups. However, the tumour incidences of the exposed animals were not increased with statistical significance compared with those of the control group (MacEwen and Vernot 1975). Unlike in the study of Toth and Shimizu (1973), the animals of this study were considerably older at the beginning of the study; this could explain why the tumour incidences were not increased.

**Tab.6:** Oral studies of the carcinogenicity of monomethylhydrazine

Author:	Toth 1972				
Substance:	monomethylhydrazine				
Species:	**mouse,** Swiss, 50 ♂, 50 ♀, control group: 110 ♂, 110 ♀				
Administration route:	oral, daily administration in the drinking water				
Concentration:	0, 0.01%, mean dose for ♂: 0.66 mg/animal and day, for ♀: 0.71 mg/animal and day (about 17.8 and 20.3 mg/kg body weight and day, respectively)				
Duration:	entire lifespan				
Toxicity:	mortality ↑				
		**Dose (mg/kg body weight and day)**			
		**0^a)^**		**♂: 17.8; ♀: 20.3**	
surviving animals	♂	1 after week 110		1 after week 70	
	♀	1 after week 120		8 after week 60	
**tumours:**					
lung adenomas	♂	10% after week 74		22% after week 51	
	♀	12% after week 90		24% after week 51	
lymphomas (malignant)	♂	2% after week 74		none	
	♀	15% after weeks 39–115		4% after week 38	
bile duct carcinomas	♂	0		1 after week 49	
	♀	0		1 after week 45	
bile duct angiomas	♂	0		6 after weeks 35, 47, 48, 51, 53 and 62	
	♀	0		2 after weeks 49 and 52	
angiomas of adrenal glands	♂	0		1 after week 61	
hepatomas	♂	0		3 after weeks 48, 51 and 61	
	♀	0		3 after weeks 59, 66 and 70	
liver angiomas	♂	2 after weeks 72 and 80		4 after weeks 43, 47, 48 and 55	
	♀	3 after weeks 69, 77 and 84		1 after week 66	
liver angiosarcomas	♂	0		2 after weeks 48 and 60	
	♀	0		1 after week 70	
liver cell carcinomas	♀	0		1 after week 67	
Author:	Toth and Shimizu 1973				
Substance:	monomethylhydrazine				
Species:	**hamster**, Syrian golden, 50 ♂, 50 ♀, control group: 100 ♂, 100 ♀				
Administration route:	oral, daily administration in the drinking water				
Concentration:	0, 0.01%, mean dose for ♂: 1.1 mg/animal and day, for ♀: 1.3 mg/animal and day (about 8.2 and 9.0 mg/kg body weight and day, respectively)				
Duration:	entire lifespan, maximum of 130 weeks				
Toxicity:	mortality ↑				
		**Dose (mg/kg body weight and day)**			
		**0**		**♂: 8.2; ♀: 9.0**	
surviving animals after 90 weeks	♂	32		8	
	♀	20		1	
**tumours:**					
histiocytomas (malignant)	♂	0		27 (54%) after week 78	
	♀	0		16 (32%) after week 70	
tumours of caecum	♂	1 after week 84		7 (14%) after week 77	
	♀	1 after week 53		9 (18%) after week 64	
polyploid adenomas in colon	♂	0		1 after week 90	
	♀	0		3 after weeks 54, 70 and 82	
dermal melanocytomas	♂	1 after week 116		0	
	♀	3 after weeks 57, 66 and 73		2 after weeks 68 and 76	
liver angiosarcomas	♀	0		2 after weeks 72 and 92	
tumours of uterine muscles	♀	3 after weeks 35, 92 and 100		2 after weeks 76 and 80	
bile duct angiomas	♀	0		1 after week 76	
hepatomas	♂	1 after week 82		0	
	♀	0		1 after week 70	
angiosarcomas of lungs and heart	♀	0		1 after week 41	
parathyroid adenomas	♀	0		1 after week 63	
liver angiomas	♀	0		1 after week 70	
carcinomas of forestomach	♂	1 after week 82		0	
	♀	0		1 after week 46	
sebaceous gland adenocarcinomas	♀	0		1 after week 76	
nerve sheath tumours (malignant)	♀	0		1 after week 64	
angiomas of fat and muscle	♀	0		1 after week 40	
papillomas of forestomach	♂	6 after weeks 66, 81, 89, 121, 124		6 after weeks 51, 67, 76, 90 and 103	
	♀	2 after weeks 80 and 92		0	
adenocarcinomas of glandular stomach	♂	0		2 after weeks 51 and 76	
adenocarcinomas of muscles of glandular stomach	♂	0		2 after weeks 76 and 80	
adrenal cortical carcinomas	♂	7 after weeks 80, 101, 111, 114, 121 and 126		2 after weeks 81 and 83	
	♀	3 after weeks 79, 94 and 110		0	
		**Dose (mg/kg body weight and day)**			
		**0**		**♂: 8.2; ♀: 9.0**	
angiomas of spleen	♂	0		1 after week 65	
carcinomas of salivary gland	♂	0		1 after week 100	
Anitschkow cell sarcomas (heart)	♂	0		1 after week 63	
squamous cell carcinomas of nasal cavity	♂	0		1 after week 47	
Author:	MacEwen and Vernot 1975				
Substance:	monomethylhydrazine				
Species:	**hamster**, Syrian golden, ♂, 30 per dose, 17 in the control group				
Administration route:	oral, daily administration in the drinking water				
Concentration:	0 (drinking water at pH 3.5), 0.01% in the drinking water without pH adjustment (7.3 mg/kg body weight and day), 0.01% monomethylhydrazine in the drinking water at pH 3.5 (7.5 mg/kg body weight and day)				
Duration:	entire lifespan				
Toxicity:	mortality ↑				
		**Dose (mg/kg body weight and day)**			
		**0 (pH 3.5)**	**7.3 (pH not adjusted)**		**7.5 (pH 3.5)**
surviving animals after 90 weeks	♂	12%	3%		0%
**tumours:**					
adenomas, carcinomas, histiocytomas, melanomas	♂	4 (31%)	4 (16%)		6 (24%)

a) data from Toth (1969)

#### 5.7.3 Summary

Although all the available studies are only of limited suitability for inclusion in the evaluation, overall, the data show that monomethylhydrazine has carcinogenic potential. In a long-term study with inhalation exposure, monomethylhydrazine induced lung adenomas, liver adenomas, liver carcinomas and haemangiomas and a low incidence of epi­thelial neoplasms in the nasal mucosa of female mice (MacEwen and Vernot 1985). Benign tumours in the nose and adrenal glands developed in male hamsters. The tumour incidences were not increased in rats or dogs. In long-term studies with oral administration (MacEwen and Vernot 1975; Toth 1972; Toth and Shimizu 1973), increased incidences of lung adenomas, bile duct angiomas and carcinomas and various liver tumours were observed in male and female mice, but these increases were not statistically significant. The latency periods for these types of tumours were shorter than those observed in the control animals. Histiocytomas and tumours in the caecum and colon developed in hamsters.

## 6 Manifesto (MAK value/classification)

Hepatotoxicity, nephrotoxicity as well as damage to the nervous system, blood and nasal mucosa are adverse effects caused by monomethylhydrazine.

**Carcinogenicity. **Exposure of workers at a rocket engine test facility to substances including monomethylhydrazine may have increased their risk of developing cancer. These data support the assumption that the substance causes carcinogenic effects in humans; however, the existing data are not sufficiently reliable to be used as valid evidence. In an inhalation study with exposure to monomethylhydrazine in concentrations of 2 to 5 ml/m^3^, lung adenomas, liver adenomas, liver carcinomas and haemangiomas and a low incidence of epithelial neoplasms of the nasal mucosa were induced in female mice. Benign tumours in the nose and adrenal glands developed in male hamsters. Increased incidences of lung adenomas, bile duct angiomas and carcinomas and various liver tumours were observed in male and female mice after oral exposure to about 25 mg/kg body weight and day, but these increases were not statistically significant. Histiocytomas and tumours in the caecum and colon developed in male and female hamsters after administration of about 10 mg/kg body weight and day in the drinking water. In vivo studies yielded evidence of a DNA-alkylating effect of the substance in the form of the binding of methyl groups to guanine. Monomethylhydrazine has been classified in Carcinogen Category 2 on the basis of the carcinogenic effects induced in 2 animal species (mouse and hamster) and the evidence of its alkylating effects on the DNA (formation of N7-methylguanine, O6-methyl­guanine and C8-methylguanine).

**MAK value. **Decreases in haemoglobin, haematocrit and the erythrocyte count were observed in rats at the low monomethylhydrazine concentration of 0.04 ml/m^3^ and above. In human studies, damage to the nasal mucosa was observed after only a single inhalation of a monomethylhydrazine concentration of 0.2 ml/m^3^; however, these studies are not sufficiently reliable for inclusion in the evaluation because they have considerable shortcomings (for example no control group). A genotoxic mode of action is of prime importance for carcinogenicity. It is not possible to establish an exposure–risk relationship. Monomethylhydrazine has not been classified in any of the peak limitation categories.

**Prenatal toxicity. **No teratogenic effects were observed after oral exposure of Sprague Dawley rats in a developmental toxicity study that was not carried out according to currently valid guidelines. As it is not possible to derive a MAK value, monomethylhydrazine has not been classified in any of the pregnancy risk groups.

**Germ cell mutagenicity. **Monomethylhydrazine is suspected of causing germ cell mutagenicity because in vitro and in vivo experiments yielded evidence of alkylating effects induced by the substance, and mutagenicity and clastogenicity were detected in somatic cells in vitro. Another reason is its structural relationship with 1,1-dimethylhydrazine and 1,2-dimethylhydrazine, which have been classified in Category 3 A for germ cell mutagens (Hartwig and MAK Commission 2024 a, b). Monomethylhydrazine has been classified in Category 3 B because there is no evidence that the germ cells are reached.

**Absorption through the skin. **Valid studies investigating the absorption of monomethylhydrazine through the skin are not available. As the dermal LD_50_ values were low, with some of them below 100 mg/kg body weight (Section 5.1.3), the “H” designation (for substances which can be absorbed through the skin in toxicologically relevant amounts) has been retained.

**Sensitization. **Monomethylhydrazine has been designated with “S” and “Sh” (for substances which cause sensitization of the skin or airways) since 1994. However, there are no data available for sensitizing effects on the skin or the respiratory tract. According to the criteria for the evaluation of sensitizing substances (see List of MAK and BAT Values, Section IV (DFG 2021)), a close structural relationship with similar substances that have been classified as sensitizing substances is not sufficient in itself to assume that the substance is likely to have a sensitizing effect if additional positive findings are not available. However, in the case of the methylhydrazines, it seems plausible that they would cause contact sensitization because of the close structural similarity to hydrazine, which is known to be a pronounced contact allergen and has been designated with “Sh”. Monomethylhydrazine was assigned the designation “Sh” in 1994 as a precautionary measure. This designation has been retained because there are no data available that demonstrate that monomethylhydrazine is not a contact allergen.
